# An influential meal: host plant dependent transcriptional variation in the beet armyworm, *Spodoptera exigua* (Lepidoptera: Noctuidae)

**DOI:** 10.1186/s12864-019-6081-7

**Published:** 2019-11-13

**Authors:** Thijmen Breeschoten, Vera I. D. Ros, M. Eric Schranz, Sabrina Simon

**Affiliations:** 1 Biosystematics Group, Wageningen University & Research, Droevendaalsesteeg 1, 6708 PB Wageningen, The Netherlands; 2 Laboratory of Virology, Wageningen University & Research, Droevendaalsesteeg 1, 6708 PB Wageningen, The Netherlands

**Keywords:** Transcriptomics, Gene expression, Detoxification, Herbivory, Generalist, Host specialization, Polyphagy, RNAseq

## Abstract

**Background:**

To understand the genetic mechanisms of insect herbivory, the transcriptional response of insects feeding on different host plant species has to be studied. Here, we generated gene expression data of the generalist herbivore *Spodoptera exigua* (Hübner) feeding on three selected host plant species and a control (artificial diet). The host plant species used in this study –cabbage (*Brassica oleracea*), maize (*Zea mays*) and tobacco (*Nicotiana tabacum*)- are members of different plant families that each employ specific defence mechanisms and toxins.

**Results:**

*Spodoptera exigua* larvae had a higher growth rate, indicator for herbivore success, when feeding on *Z. mays* compared to larvae feeding on *B. oleracea* or *N. tabacum*. Larvae feeding on the different host plant species showed divergent transcriptional responses. We identified shared and unique gene expression patterns dependent of the host plant species the larvae fed on. Unique gene expression patterns, containing uniquely upregulated transcripts including specific detoxification genes, were found for larvae feeding on either *B. oleracea* or *N. tabacum*. No diet-specific gene cluster was identified for larvae feeding on the host for which larvae showed optimal herbivore success, *Z. mays,* or artificial diet*.* In contrast, for larvae feeding on hosts for which they showed low herbivore success, specific diet-dependent gene clusters were identified. Functional annotation of these clusters indicates that *S. exigua* larvae deploy particular host plant-specific genes for digestion and detoxification.

**Conclusions:**

The lack of a host plant-specific gene activity for larvae feeding on *Z. mays* and the artificial diet suggest a general and non-specific gene activity for host plants with optimal herbivore success. Whereas the finding of specific gene clusters containing particular digestion and detoxifying genes expressed in larvae feeding on *B. oleracea* and *N. tabacum*, with low herbivore success, imply a host plant-specific gene activity for larvae feeding on host plants with suboptimal herbivore success. This observation leads to the conclusion that a polyphagous herbivore is able to feed on a large variation of host plants due to the flexibility and diversity of genes involved in digestion and detoxification that are deployed in response to particular host plant species.

## Background

The 400 million years of interaction and co-evolution between plants and insects has led to a wide diversity of plant defences, to which herbivorous insects in turn have evolved a diverse array of resistance and detoxification mechanisms [1]. Numerous herbivorous insects have evolved the ability to feed on a large range of host plant species (polyphagy), thereby encountering a variety of plant-specific defence compounds [2, 3]. The ability of an herbivorous insect to feed on different host plants does not imply equal herbivore success on each of these plants. This success is partly dependent on nutrient content and plant defence resistance and is reflected by growth- and developmental rate of the insect [4, 5]. In herbivorous insects the detoxification of plant defence compounds follow a three phased pathway involving members of several known enzyme families. In phase I of the detoxification process, cytochrome P450 monooxygenases (P450s) and carboxyl/choline esterases (CCEs) metabolize toxins [1]. The metabolized product is conjugated by phase II enzymes such as UDP-glycosyltransferases (UGTs) and glutathione-S-transferases (GSTs), and transported out of the cell by transporters like ATP-binding cassettes (ABCs) and solute carrier proteins (SLC) in phase III [1, 6, 7].

The ability of an herbivorous insect to feed on a broad host range largely depends on the flexibility and diversity of the insect’s digestion and detoxification system. A recent comparative genomic study by Pearce et al. [8] showed major expansions of gene families involved in detoxification and digestion including P450s, GSTs and CCEs when comparing two polyphagous moth species to two monophagous species. The evolution of polyphagy is hypothesized to be associated with expansions of gene families involved in host plant use, due to gene duplication and neofunctionalisation, (e.g. [8–11]). Indeed, expansions of detoxification and digestion related gene families have been observed in multiple polyphagous arthropod species, such as the spider mite (*Tetranychus urticae*), known to feed on over 1000 different host plant species [11–13], the tobacco cutworm (*Spodoptera litura*) [10], the fall armyworm (*Spodoptera frugiperda*) [14] or the whitefly (*Bemisia tabaci*) [15].

Empirical evidence for the role of genes involved in the detoxifying ability of insects is mainly based on experimental studies using pesticide and isolated toxin treatments (e.g. [10, 16–18]). More recently, plants are incorporated into molecular studies on the transcriptional response of insects, mimicking a more natural system. These studies have shown a differential genetic response of insects after host plant switches [19], or the transcriptional response of feeding on specific host plants [20–23]. Yet, a multi-comparison of the genetic response of a polyphagous insect on multiple hosts from diverse plant families would provide insights in shared and unique gene activity linked to specific host plant usage.

**Fig. 1:**
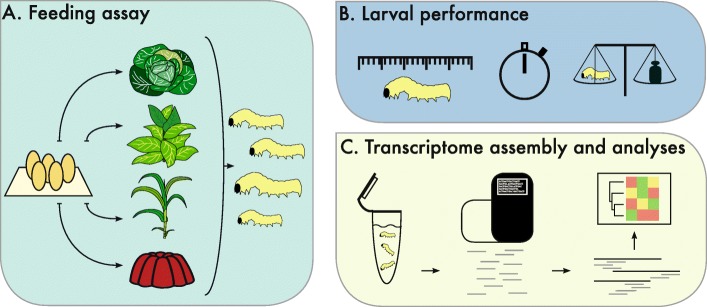
Overview of the experimental design to study the gene expression differences in *Spodoptera exigua* larvae feeding on different host plants. **a** Feeding assay: larvae hatched and developed on three different host plants (*Brassica oleracea, Nicotiana tabacum* and *Zea mays*) and artificial diet as control. **b** larval weight and developmental time was recorded to quantify herbivore success (growth rate in mg/day). **c** RNAseq data was generated of each diet treatment, followed by de novo assembly and differential gene expression analyses

In the present study we analysed the gene expression of the polyphagous beet armyworm, *Spodoptera exigua* (Hübner), feeding and developing on three of its recorded host plants and artificial diet (Fig. 1a). This species is a member of the family Noctuidae and occurs worldwide except for cold regions [24]. *Spodoptera exigua* is a polyphagous herbivore being able to accept over 130 host plants representing more than 30 families [4, 25]. Many of these host plants are considered economically important crops, making *S. exigua* an agricultural pest species [5, 26, 27]. In our study we used three host plant species: cabbage (*Brassica oleracea*), maize (*Zea mays*) and tobacco (*Nicotiana tabacum*). They are members of three distantly related families: the crucifers (Brassicaceae), the grasses (Poaceae) and the nightshades (Solanaceae), respectively, and employ different defence mechanisms. The diverse plant families, as represented by the selected host species in the experimental comparison, are known for their specific defence compounds: *B. oleracea* produces glucosinolates, which are most dominant in the Brassicaceae family [28], *Z. mays* produces benzoxazinoids [29, 30] and *N. tabacum* produces various alkaloids including nicotine [31]. We aimed to identify shared and/or unique gene expression patterns in relation to the different host plants. The identification of these expression patterns provides us information on the general genetic mechanism of herbivory, and, moreover, shows the diversity in transcriptional responses of insects while feeding on alternative host plants. Eventually, this is of importance in the understanding of the evolutionary and molecular mechanisms of herbivory as a whole. We analysed larval performance after feeding assays on the selected host plant species to quantify herbivore success and adaptability to the specific host defences (Fig. 1b). Furthermore, we studied and compared the transcriptional response of *S. exigua* larvae feeding on the host plants using RNAseq (Fig. 1c). We identified differential gene expression patterns as a result of feeding on different hosts.

Our study revealed unique clusters of genes with a diet-dependent expression in larvae feeding on hosts with sub-optimal herbivore success: *B. oleracea* and *N. tabacum.* A specific expression pattern containing uniquely expressed genes was not observed for larvae feeding on *Z. mays*, for which the feeding assay showed optimal herbivore success.

## Results

### Feeding assays

**Fig. 2:**
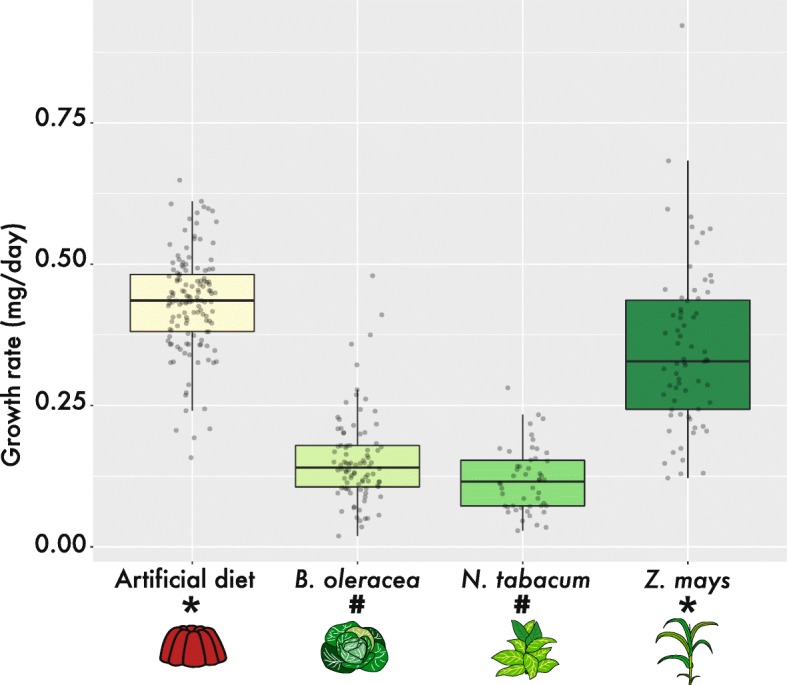
Graph showing the growth rates of *Spodoptera exigua* larvae collected in the third larval stage developing on different hosts, *Zea mays*, *Brassica oleracea, Nicotiana tabacum* and artificial diet. Growth rate (mg/day) was calculated by dividing the total larval weight (mg) by the number of days the larvae were allowed to feed (point of collecting: first observed larva reaching third larval phase). Asterisks (*) indicate significant different treatments compared to all others. The hashtag (#) indicate treatments that are not significantly different from each other. The graph was created with the R function ggplot2 [87] with spacing between datapoints

Herbivore success of *S. exigua* larvae on the different host plant species was assessed in feeding assays comparing growth rates (weight/developmental time). The weight differences and the growth rates (mg/day) between the diet treatments were significantly different (ANOVA, *p*-value <1e^− 16^). Larvae on *Z. mays* proved to have the highest herbivore success (Fig. 2; *N* = 68; weight: 2.103 ± 0.56 mg; growth rate: 0.35 ± 0.15 (mean ± SD) mg/day; developmental time: 6 days) both the weight and growth rate are significantly higher compared to all other host plant treatments as calculated with a post-hoc Dunn-test. The weight and growth rates of larvae developing on *B. oleracea* (*N* = 92; weight: 1.08 ± 0.87 mg; growth rate: 0.154 ± 0.08 mg/day; developmental time: 7 days) were not significantly different from larvae developing on *N. tabacum*, under the assay conditions as described in the methods part (*N* = 50; weight: 1.065 ± 0.51 mg; growth rate: 0.118 ± 0.06 mg/day; developmental time: 9 days). Larvae developing on the artificial diet (*N* = 134; weight: 2.162 ± 0.44 mg; growth rate: 0.433 ± 0.09 mg/day; developmental time: 5 days) had significantly the highest growth rate as compared to all other diets but larval weight was not significantly different from larvae feeding on *Z. mays*. See Additional file 1: Table S1 for details of feeding assay results and Additional file 2: Table S2 for results of the feeding assay statistics*.*

### Transcriptome assembly

For each of the four treatments three replicates consisting of five larvae each were created. The 12 RNA libraries were sequenced on an Illumina HiSeq platform and the raw reads were assembled using Trinity v.2.5.1 [32]. See Additional file 12: Data S1 for a description of the transcriptome assembly statistics and Additional file 3: Table S3 for a total overview of the number of raw reads per library and the final number of reads after trimming, cleaning and contamination checks.

The assembly has been submitted to NCBI TSA database (GGRZ00000000) and has been used as a reference for the transcript expression quantification.

A completeness analysis of the final assembly using BUSCO v.3.0.2 against the *Insecta* gene set [33] indicated a high completeness level of expressed genes of 97.3%, with 1088 complete single-copy BUSCOs, 526 complete duplicated BUSCOs, 21 fragmented BUSCOs and 23 missing BUSCOs of the in total 1658 total BUSCO groups that were searched.

### Transcript expression quantification

**Fig. 3:**
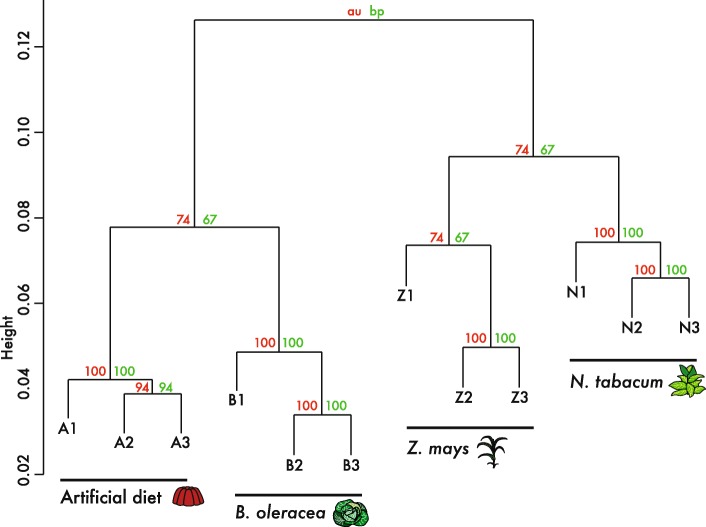
Hierarchical cluster dendrogram using multiscale bootstrap resampling of *Spodoptera exigua* larvae samples with different diet treatments each consisting of three replicates. The filtered (gene count of ≥10 and occurrence ≥2 samples) and normalized (CPM + TMM normalization) gene count matrix was used as input, including 58,749 genes. The number of bootstrap replications was 10,000. Given are Bootstrap Probability (BP) and the Approximately Unbiased (AU) values based on complete hierarchical clustering using the correlation distance measure

The R package PVClust v.2.0 [34] was used to analyse the sample relationships based on the filtered and normalised count matrix. The 10,000 bootstrap replicate based hierarchical clustering showed that replicates of the different diet treatment were more similar to each other than to samples of other treatments (Fig. 3), proving the differentiation of the samples according to diet. Sample Z1 within the *Z. mays* diet treatments did show increased variation with the other samples compared to all other treatments but was forming a cluster with the other maize replicates based on the PVClust analysis. The expression pattern of larvae feeding on *Z. mays* was most closely resembling the *N. tabacum* diet treatments. Furthermore, *B. oleracea* diet treatments clustered together with larvae of the control group feeding on the artificial diet, while *Z. mays* diet treatments clustered together with *N. tabacum* diet treatments.

We have used the trinity pipeline to perform a differential gene expression analysis with DESeq2 v.1.18.1 [35] to identify transcripts differentially expressed (DE) across samples. Transcripts were considered DE with a fold change of 2^2^ and *p*-value ≤1e^− 3^.

**Fig. 4:**
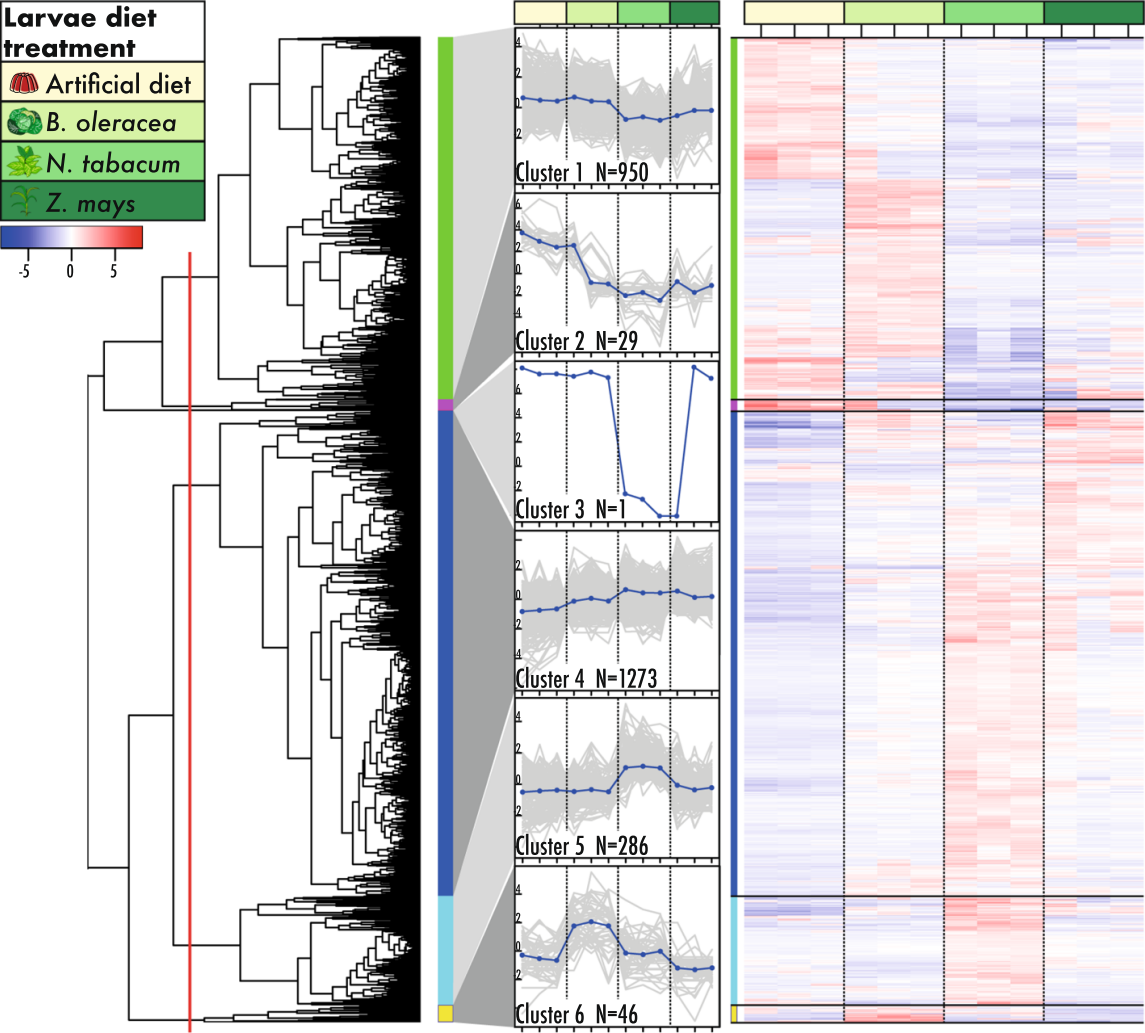
Hierarchical clustering dendrogram of all differentially expressed genes (DE) (left) in *Spodoptera exigua* larvae with different diet treatments clusters the 2585 DE in 6 clusters using a cut-of at 50% (red line). Expression patterns were visualized and the number of genes (N = #) per cluster is indicated (middle). Green colour coding indicates host diet treatment, larvae fed with *B. oleracea, N. tabacum* and *Z. mays* are shown with increasing darker shades of green. Each diet treatment consisted of three replicates. The heatmap (right) shows the expression pattern of the DE genes, black lines indicate the cluster devision. Each column corresponds to larvae from different diet treatments as indicated by the colour scheme

The transcripts were clustered based on the expression patterns. A cut-off of 50% for the hierarchical-clustering dendrogram was used. This resulted in the 2585 DE genes being clustered into six groups (graphical representation and visualisation of the expression patterns of the DE genes and differences between the diet treatments, Fig. 4). In two clusters a treatment-dependent gene expression was observed. Cluster 5 consisted of 286 transcripts that were upregulated in the *N. tabacum* treatment. Cluster 6 consisted of 46 transcripts upregulated in larvae feeding on *B. oleracea.* Transcripts in cluster 2 and 3 showed upregulation in samples from multiple treatments. Upregulation of genes in multiple treatments and/or absence of a response in part of the samples within a treatment are interpreted as non-host plant species-specific gene expression effects. Cluster 2 consisted of 29 transcripts that showed upregulation in all samples from the control group and a single sample from the *B. oleracea* treatment. Cluster 3 consisted of a single transcript only, downregulated in *N. tabacum* samples and a *Z. mays* sample. Cluster 1 and 4 contained the highest number of transcripts. Cluster 1 consisted of 950 transcripts that showed upregulation in larvae feeding on the artificial diet and *B. oleracea*. Cluster 4 consisted of 1273 transcripts with upregulation in all host plant diet treatments (Fig. 4).

### Transcript annotation

The reference transcriptome assembly was annotated using the Trinotate pipeline v.3.0 [36] (See Additional file 4: Table S4 for the Trinotate annotation report). Additionally all DE transcripts were used as a query in a local BLASTX search against a local database containing all Arthropoda protein sequences as retrieved from the NCBI protein database (see Additional file 5: Table S5 for the BLASTX annotated DE transcripts using the local Arthropoda database).

Of the 2586 DE genes 940 retrieved a GO annotation. Both cluster 3 (transcript downregulated in *N. tabacum* samples and a *Z. mays* sample) and cluster 6 (transcripts upregulated in larvae feeding on *B. oleracea*) had no significant GO terms overrepresented. The GO terms that showed statistical enrichment in the remaining clusters were compiled into broader generic GO slim categories for the ontology categories of biological processes (BP) and molecular functions (MF). Since the majority of the MF terms were summarized into the GO slim ‘molecular function’ (GO:0003674), GO terms were additionally compiled into the child terms of the GO slim ‘molecular function’, to increase specificity. GO terms of cluster 1 (transcripts upregulated in larvae feeding on the artificial diet and *B. oleracea*) and cluster 4 (transcripts upregulated in all host plant diet treatments) were summarized into both BP and MF category GO slims while for cluster 2 (transcripts upregulated in control samples and a *B. oleracea* sample) and cluster 5 (transcripts upregulated in the *N. tabacum* treatment) GO slims were only retrieved from the MF category. See Additional file 6: Table S6 for an overview of all GO slims per cluster.

Major represented GO terms according to the BP GO slims in cluster 1 (transcripts upregulated in larvae feeding on the artificial diet and *B. oleracea*) were ‘immune system process’ (GO:0002376), ‘response to stress’ (GO:0006950), ‘signal transduction’ (GO:0007165), ‘reproduction’ (GO:0000003) and ‘catabolic process’ (GO:0009056). Major MF GO slims were ‘peptidase activity’ (GO:0008233), ‘oxidoreductase activity’ (GO:0016491) and ‘molecular function’ (GO:0003674) with major significant child terms of the GO slim ‘molecular function’: ‘catalytic activity’ (GO:0003824), ‘binding’ (GO:0005488) and ‘structural molecule activity’ (GO:0005198).

In cluster 4 (transcripts upregulated in all host plant diet treatments) major GO slims were ‘circulatory system process’ (GO:0003013), ‘reproduction’ (GO:0000003) and ‘nervous system process’ (GO:0050877) for the BP category. Major MF GO slims were ‘transmembrane transporter activity’ (GO:0022857) and ‘molecular function’ (GO:0003674) with major significant child terms of the GO slim ‘molecular function’: ‘transporter activity’ (GO:0005215), ‘catalytic activity’ (GO:0003824), ‘binding’ (GO:0005488) and ‘molecular transducer activity’ (GO:0060089).

For cluster 5 (transcripts upregulated in the *N. tabacum* treatment) MF GO slims of this cluster were ‘oxidoreductase activity’ (GO:0016491), ‘transferase activity, (alkyl/aryl groups)’ (GO:0016765), ‘ion binding’ (GO:0043167) and ‘molecular function’ (GO:0003674) of which the significant child terms were grouped in the GO slims ‘catalytic activity’ (GO:0003824) and ‘binding’ (GO:0005488). For cluster 6 (transcripts upregulated in larvae feeding on *B. oleracea*) no significant overrepresented GO terms were present.

### Detoxification genes

The reference transcriptome was further analysed for putative detoxification genes of five major selected gene families: Cytochrome P450s (P450), carboxyl/cholinesterases (CCE), glutathione S-transferases (GST), UDP-glycosyltransferases (UGT) and ATP-binding cassette transporters (ABC transporters). In total, 210,101 transcripts were searched of which 3772 transcripts were annotated as putative detoxification genes from one of the five major selected gene families. In total 208 putative detoxification genes were DE (fold change of 2^2^, *p*-value ≤1e^− 3^). The majority of transcripts in the set of DE transcripts were members of the ABC transporter enzyme family (75 transcripts). The four other gene families were less often identified: P450 (51 transcripts), GST (26 transcripts), CCE (37 transcripts), and UGT (19 transcripts). Each cluster, except for cluster 3, contained multiple annotated transcripts identified as genes from one or multiple detoxification families. In cluster 1 (transcripts upregulated in larvae feeding on the artificial diet and *B. oleracea;* 66 annotated transcripts), cluster 4 (transcripts upregulated in all host plant diet treatments; 91 identified transcripts) and cluster 5 (transcripts upregulated in the *N. tabacum* treatment; 41 annotated transcripts) all five detoxification families were present. Cluster 2 (transcripts upregulated in control samples and a *B. oleracea* sample; 4 annotated transcripts in total) only contained ABC genes. In cluster 6 (transcripts upregulated in larvae feeding on *B. oleracea*) in total six transcripts were identified from the ABC, CCE and GST families. See Additional file 7: Table S7 for the BLASTX annotated DE genes using the local detoxification gene database and Table 1 for an overview of transcripts identified as detoxification genes per gene expression cluster.

**Table 1:** Transcripts annotated as detoxification gene family per gene expression cluster

Cluster	Gene family	Group/Clade/Clan	#
Cluster 1	P450	clan-3	4
		clan-4	2
	UGT	UGT33	7
		UGT34	1
		UGT39	1
	GST	Sigma	3
	CCE		10
	ABC	ABCA	24
		ABCB	2
		ABCC	2
		ABCD	9
		ABCG	1
Cluster 2	ABC	ABCA	4
Cluster 3	–		
Cluster 4	P450	clan-2	1
		clan-3	21
		clan-4	11
		clan-M	1
	UGT	UGT33	4
		UGT39	1
		UGT40	3
	GST	Sigma	1
		Delta	1
		Omega	2
		Other/ Unknown	7
	CCE		15
	ABC	ABCA	6
		ABCD	11
		ABCG	7
Cluster 5	P450	clan-2	1
		clan-3	8
		clan-4	2
	UGT	UGT40	2
	GST	Sigma	3
		Other/ Unknown	9
	CCE		8
	ABC	ABCB	2
		ABCC	1
		ABCD	2
		ABCF	1
		ABCG	2
Cluster 6	GST	Other/ Unknown	1
	CCE		4
	ABC	ABCG	1

## Discussion

We compared the gene expression patterns of larvae of the polyphagous beet armyworm, *Spodoptera exigua* (Hübner) feeding and developing on different host plant species. We aimed to identify shared and/or unique gene expression patterns to study the general genetic mechanisms of herbivory and observe transcriptional response due to host plant differences. All treatment specific results are due to experimental setup design host plant specific and are thus diet-dependent.

Given the highest growth rates in larvae feeding on *Z. mays* compared to the other host plant species indicate that *S. exigua* larvae from this population were better able to utilize the nutrients available in *Z. mays* and/or cope with the specific defences. Diet treatment-dependent gene clusters with uniquely upregulated genes, including cluster-specific detoxification and digestion related genes, were only found in larvae feeding on *B. oleracea* or *N. tabacum.* This observation shows that *S. exigua*, considered as a major polyphagous insect, relies on the deployment of specific genes against particular host plant species. Specimens of *S. exigua* as used in this study originated from a lab culture recurrently refreshed with individuals from other populations. Different populations of generalist insects often demonstrate certain degrees of host specialisation on a local level [2]. By using a lab population, both the background and feeding history is known which is important for studying the gene plasticity of feeding on different host plant species. Previous studies have shown that lab populations without regular renewal of individuals do adapt to their specific rearing conditions [45]. The strain of *S. exigua* was kept on an artificial diet, not resembling the live plant material the larvae are exposed to during the feeding assays. The artificial diet does contain plant-based products derived from seeds but is heavily produced and does not contain (whole or detached) plant parts such as leaves. Here we identify the transcriptional responses of polyphagy within this population of *S. exigua*. Comparing wild local populations is an interesting next step for understanding within-species differences.

The selected host plants are distantly related and produce a variety of different secondary metabolites for defence: *Z. mays:* benzoxazinoids, *B. oleracea*: glucosinolates and *N. tabacum:* various alkaloids like nicotine [46]. *Spodoptera exigua* is considered a major polyphagous species, being able to feed and develop on a large variety of host plants [25]. However, the feeding assay indicates a different degree of host plant usage and response to the different plant defences. As expected, larvae feeding on artificial diet showed an optimal development with the highest weight gain per day in comparison to larvae feeding on any of the host plants. The artificial diet is optimal in nutrient content and lack of any herbivory defences, like secondary metabolites. In addition, previous generations have been reared on the artificial diet in the stock rearing (see Methods section).

For the *N. tabacum* feeding assay a slightly adjusted experimental design has been used with an initial feeding period on detached leaves preceding a transfer to full plants (see for details Methods section). This adjusted treatment was used to lower larval mortality in the first larval phase on *N. tabacum*. These larvae have not been exposed to induced plant defences in response to herbivory as larvae feeding on the other plants had. Still, larvae were exposed to the defences present in the host plant during collecting. Plants alter physical and chemical defensive mechanisms upon herbivory [1, 47, 48]. The increased defences of *N. tabacum* might have caused the high mortality during the critical first larval stage [5]. In initial trials all larvae feeding on full plants of *N. tabacum* died within 24 h after hatching. Providing larvae detached leaves during the first larval stage prevented the possibility of the *N. tabacum* plant to increase defense compounds. Yet, transferring the larvae back on the full plant in the second larval stage is important to restore induced interactions [49]. Providing first instar larvae detached leaves proved necessary due to high mortality. The interplay between induced defences of living plants in response of feeding insects shows the importance of using full living plants in studies analysing plant-insect interactions.

### Analysis of DE transcripts

The differential gene analysis showed that gene expression in larvae of *S. exigua* is dependent on the host plant*.* Two out of the six clusters showed upregulation of transcripts for only a single host-plant; cluster 5 for *N. tabacum* and cluster 6 for *B. oleracea*. Interestingly, such a host-specific gene expression activity pattern is missing for the other two diet types where *S. exigua* larvae showed optimal levels of herbivory: artificial diet and the host plant *Z. mays*.

Transcripts in cluster 1 showed upregulation in larvae with the artificial diet and *B. oleracea* diet treatments. Most GO terms were summarized into the GO slim ‘immune system process’(GO:0002376) which includes genes involved in the development and role in the immune system. Various transcripts are identified as antimicrobial peptides including attacin (three transcripts), gloverin (one transcript) and cecropin (three transcripts) involved in acting against microbial intrusion in the hemolymph by targeting the microbial membrane [50, 51]. Another group of proteins involved in the immune system response and identified within cluster 1 are various REPAT proteins. Although cluster 2 and 6 both contained unique REPAT genes as well (cluster 2: REPAT 45, cluster 6: REPAT 10 and 12), cluster 1 contained 20 transcripts identified as REPAT from 16 different REPAT types. REPAT genes showed increased expression in response to pathogens inside the midgut [52]. However, it is speculated that besides this response to pathogens part of the REPAT proteins are involved in different physiological processes of the midgut [53].

The expression profile of cluster 4 included transcripts with upregulation in all plant diet treatments as compared to the artificial diet. Among the transcripts annotated in this cluster were 39 (BLASTX identification; 41 with Trinotate) encoding for cuticle-related proteins. An upregulation of genes involved in cuticle formation can be an indication for diet response. The insect cuticle and peritrophic matrix of the midgut serve in the correct functioning and movement of these organs [54]. Being in direct contact with abrasive food particles, the peritrophic matrix forms a biochemical and physical barrier and is involved in toxin inactivation [55]. Alteration of the cuticle protein composition can lead to a more resilient and protective cuticle [56, 57]. Similarly to cluster 4, multiple transcripts in cluster 1 (nine according to BLASTX annotation; 10 according to Trinotate annotation) are identified as being involved in cuticle development. This cluster shows upregulation of transcripts in larvae fed with artificial diet and *B. oleracea*. Indeed, both clusters 1 and 4 are characterised by GO slims linked to the development of the cuticle, e.g. ‘anatomical structure development’. Yet, the higher number of cuticle-related genes in cluster 4, upregulated in larvae fed on host plants, indicates that this mechanism might play a general role in coping with the various plant defence toxins. The overrepresentation of chitin related genes is not unique for *S. exigua* and has been found in other Noctuidae species with different host plants (e.g. [21, 23]).

We further focussed on the gene activity of five major detoxification gene families. This is of special interest for gene clusters showing upregulation in larvae feeding on a single host plant like clusters 5 and 6. Genes uniquely expressed in a single diet treatment are indicative for a potential host-specific response, e.g. detoxification genes acting specifically on alkaloids, the defensive compounds of *N. tabacum*. Members of all five detoxification families were present in cluster 5, with transcripts upregulated in larvae feeding on *N. tabacum.* Cytochrome P450 monooxygenases are involved in metabolizing toxins in the first phase of the detoxification pathway. In cluster 5, 11 P450 genes were identified of which 10 were found uniquely expressed in this cluster. The majority of the genes are clade-3 P450s, well known for their role in the detoxification of plant defence secondary metabolites (e.g. [9, 58, 59]). The specific expression of the identified CYP6AE and CYP321 members within larvae feeding on *N. tabacum* might indicate a role in alkaloid detoxification. This confirms recent findings for the role of these genes in xenobiotic detoxification within the cotton bollworm (*Helicoverpa armigera*) and the tobacco cutworm (*Spodoptera litura*) [60–62]. Of the eight transcripts identified as carboxyl/choline esterases that also act in the first detoxification phase, two genes were uniquely expressed in this cluster. CCEs have been recognized as an important component of xenobiotic detoxification in insects [63]. Expansions of the gene families in polyphagous Noctuidae suggest a link with increased detoxification ability [8, 10].

Both UDP-glycosyltransferases and glutathione-S-transferases are active in the second phase of the detoxification process. Of both families gene members have been identified in cluster 5. The two transcripts annotated as UGT proteins were both identified as UGT40U2. Members of the UGT40 family have been found particularly upregulated in digestion and detoxification-related body parts of *Helicoverpa* species [8]. Tissue-specific expression profiles of UGT genes in *S. exigua* showed high expression of UGT40U2 in the fat body of larvae [18]. However, this gene is also present in cluster 4 showing increased expression in all host treatments suggesting a broad gene activity.

Moreover, we identified 12 transcripts in cluster 5 as GST genes; one of these was uniquely expressed in larvae feeding on *N. tabacum*. This GST gene is a member of the microsomal subfamily that is thought to be involved in oxidative stress response [64, 65].

ABC transporters are active in the third phase of detoxification. ABCs are involved in the transport of different compounds, many members show increased activity upon xenobiotic treatment ([10, 66]; and references therein). ABCs involved in xenobiotic detoxification processes have been identified to belong to the ABC-B, ABC-C and ABC-G subfamilies [67, 68]. Eight transcripts were identified as ABC genes within cluster 5. Only two ABC genes were uniquely expressed in the *N. tabacum* diet treatment samples: ABC-C2 and ABC-F4. ABC-C2 has been identified as the receptor of several specific Bt (Cry) toxins, a widely used biopesticide, in many Lepidoptera taxa including *S. exigua* [69–71]*.* ABC-C2 is only upregulated in larvae feeding on *N. tabacum* absent of Cry proteins, suggesting that ABC-C2 only has a role in alkaloid resistance.

All transcripts grouped in cluster 6 showed upregulation in larvae feeding on *B. oleracea.* Genes of only three out of five detoxification families were identified: CCE, GST and ABC transporters. *B. oleracea* uses glucosinolates as defence, in contrast to the other hosts. The detoxification genes grouped within this cluster might act upon glucosinolate presence [72]. The detoxification genes upregulated in larvae feeding on a specific host, *N. tabacum* in cluster 5 and *B. oleracea* in cluster 6, are indicative for a host-specific role in detoxification. Detoxification genes identified in clusters with stable expression across hosts suggests a general physiological role or broad toxin acceptance.

In addition to detoxification-related genes in cluster 6, transcripts involved in the general digestion of plant material have been annotated. Three transcripts are annotated as trypsins, which together with chymotrypsins are involved in the digestion of proteins. The trypsins in cluster 6, according to the BLASTX annotation are of the alkaline c-type. Furthermore, alkaline c-type trypsins have been identified in the clusters 1, 3 and 4. Trypsins play an important role in insect survival. Various *Nicotiana* species successfully use trypsin inhibitors as defence against insect herbivores [73]. But insects evolved adaptive responses [74]. In all clusters trypsins and/or chymotrypsins have been identified but they are absent in cluster 5, showing upregulation of genes in larvae feeding on *N. tabacum*.

In summary, the identified gene expression clusters show the transcriptional response of *S. exigua* larvae feeding on different host plants that utilize species-specific defence mechanisms. The results of the feeding assays, quantifying the herbivore success for each host, showed that larvae of the beet armyworm had the highest growth rate when feeding on *Z. mays* compared to *B. oleracea* and *N. tabacum* fed larvae. This indicates that the used population of *S. exigua* is better able to cope with the *Z. mays-*specific plant defences and conversion of the material into biomass in comparison to the other host plant species. We found six clusters of transcripts differing in their expression pattern. Clusters with genes upregulated in a single diet treatment were observed for larvae feeding on either *B. oleracea* or *N. tabacum*, the hosts for which larvae showed low herbivore success. Among the genes uniquely upregulated in either *B. oleracea* or *N. tabacum* diet treatments were multiple members from major gene families putatively involved in plant toxin detoxification: P450 monooxygenases, carboxyl/choline esterases, UDP-glycosyltransferases, glutathione-S-transferases and ABC-transporters. No specific diet-dependent gene clusters were identified for larvae feeding on the diet treatments with optimal herbivore success, *Z. mays* and the artificial diet. This indicates that for *S. exigua* larvae feeding on host plant species under optimal growth rates a general gene activity is used in the digestion and detoxification of the plant material. On the contrary, *S. exigua* larvae feeding on host plants with low herbivore success deploy particular host plant-specific (detoxification) genes for digestion and survival.

## Conclusions

Our work shows how a major polyphagous insect relies on the deployment of various host plant-specific genes. The importance of diverse and flexible gene expression involved in digestion and detoxification for a major polyphagous herbivore is evident from this study. We conclude that a generalist herbivore like *S. exigua* deploys a flexible set of genes in order to be able to survive on a wide array of host plant species.

This work is based on the gene activity of a single species. Clearly, comparative transcriptomic studies of related polyphagous insect species using a similar set-up are needed to verify this finding and elucidate the gene activity of major polyphagous herbivore insects feeding on different host plants. Moreover, the increasing amount of genomic data makes large-scale comparative work possible to study and understand the molecular and evolutionary mechanisms of insect herbivory.

## Methods

### Host plants and *Spodoptera exigua* rearing

*Spodoptera exigua* specimens originated from a stock rearing of the Laboratory of Virology, Wageningen University & Research, kept on artificial diet at 27 °C with 50% relative humidity and a 14:10 h light:dark photoperiod. This population is recurrently renewed with specimens from other populations. The artificial diet consisted of water, corn flower, agar, yeast, wheat germ, sorbic acid, methylparaben, ascorbic acid and streptomycin sulphate. In the stock rearing, adult moths were kept in cylindrical containers, lined with paper sheets for egg deposition.

For the host plant experiments three different plant species were used in this study: *Zea mays* L. (accession B73, PI550473 –lot 94ncai02; propagated by selfing at the University of Amsterdam, seeds provided by Dr. M.E. Stam), *Brassica oleracea var. gemmifera* L. cultivar Cyrus (provided by Unifarm Wageningen University & Research, seeds from Syngenta Seeds, The Netherlands), and *Nicotiana tabacum* L. (accession TC325, PI552514; provided by Dr. J.M. Nifong, US Nicotiana Germplasm Collection). Plants were sowed and grown under optimal species-specific conditions at the Unifarm Wageningen University & Research greenhouse facilities until use in feeding assays.

### Feeding assays

To quantify the success of herbivory, larval performance was recorded in feeding assays. Feeding assays consisted of larvae developing and feeding on a single individual host plant, which was placed in a separate breeding cage, or on artificial diet in a plastic container (control group). The feeding assays took place in a temperature controlled greenhouse compartment at Wageningen University & Research (18 °C:20 °C, controlled lowest minimum night:day temperature; and 16:8 light:dark photoperiod). Larvae never had a lack of food nor were they reared under high population densities to avoid aggression and cannibalism [75].

Multiple egg clutches were selected counting up to a total of ±300 eggs using a dissection microscope and eggs were placed on top of leaves of whole plants inside the cages, or in plastic containers with artificial diet (control). The plants used for feeding assays were 5–9 weeks old. Hatched larvae were allowed to feed on the host plant or artificial diet until reaching the third larval stage. As soon as the first larva reached the third larval stage all larvae were collected on ice, frozen and weighed on a Sartorius MSE3.6P-000-DM Cubis Micro Balance (Sartorius, Göttingen, Germany). The collection point was chosen to have a similar time point for all treatments, based on development. The growth rate per day up to larval collection (mg/day) was calculated for each individual larva by dividing the larval weights by the recorded developmental time (days passed from hatching until larvae collecting).

Due to 100% mortality of larvae during the first larval stage after hatching on whole living *N. tabacum* plants, we followed a slightly modified experimental design for this host plant: eggs were placed on detached leaves and the larvae were allowed to develop on detached leaves under controlled conditions (27 °C, 50% humidity, 14:10 h light:dark photoperiod) until reaching the late-second larval stage. This approach ensured larvae were facing the specific plant material at all times but without increased levels of induced defences at the more sensitive early stage. Afterwards larvae were relocated to the whole plant set-up, restoring the natural plant-herbivore interactions. Larvae then moulted to the third larval stage and continued feeding; solely larvae with proven feeding (close-by feeding damage visible on the plant) were collected after 48 h.

The weight and growth rates of all four different diet treatments were processed and checked for significance using a non-parametric one-way ANOVA test followed by a Dunn-test for pairwise comparison using the Bonferroni method for *p*-value adjustment in R v.3.4.3.

### Larvae rearing for RNAseq

Larvae used for the RNA sequencing were treated exactly similar as larvae in the feeding assays.

Larvae were collected on the first day of reaching the third larval stage, directly ground and frozen in RNAlater reagent (QIAGEN, Hilden, Germany) and kept at − 80 °C until RNA isolation. Only larvae in the third larval stage were included. Morphological characteristics were used to confirm the correct larval stage: at end of L2 stage black stripe is visible, head of freshly moulted larvae is as wide as its body and the spiracular line becomes visible. For each treatment three biological replicates of five larvae each were created, based on initial survival tests.

### RNA sequencing

All samples for RNAseq were sent to Novogene (Novogene Co., Ltd.) for RNA extraction, cDNA library preparation and sequencing. Total RNA was extracted from each individual sample using TRIzol Reagent following the manufacturer’s instructions (Invitrogen Co. Ltd., San Diego, USA). RNA degradation was checked for all samples on 1% agarose gel electrophoresis. The quality and integrity of the RNA samples was checked using the Nano 6000 Assay Kit of the Bioanalyzer 2100 (Agilent technologies, CA, USA) and the NanoPhotometer spectrophotometer (Implen, CA, USA). The RNA concentration was measured using the Qubit RNA Assay Kit on a Qubit 2.0 Fluorometer (Invitrogen Co. Ltd., San Diego, USA). RNA concentrations are given in Additional file 8: Table S8, indicating the quality and consistency of the RNA samples used.

The NEBNext Ultra RNA Library Prep Kit for Illumina sequencing (NEB, MA, USA) following the protocol as provided by the manufacturer was used for library preparation, index sequences were added to trace back the sequences to each sample. In short, a total of 3 μg total RNA extract was used for each library preparation. The mRNA was purified using poly-T oligo-attached magnetic beads followed by fragmentation using NEBNext First Strand Synthesis Reaction Buffer (5x). Synthesis of first strand cDNA was performed using random hexamer primers and M-MuLV Reverse Transcriptase (RNase H^−^), subsequently second strand cDNA was synthesized using DNA polymerase I and RNase H. After terminal repair of the sequences, the 3′ ends were adenylated and NEBnext adapters (NEB, MA, USA) were ligated. The library fragments were purified, for a 150–200 bp size range, with AMpure XP purification kit (Beckman Coulter, MA, USA). To the size-selected cDNA, 3 μl USER Enzyme (NEB, MA, USA) was added and heated to 37 °C for 15 min followed by 5 min at 95 °C. The cDNA was enriched by PCR reactions using Phusion High-Fidelity DNA polymerase, universal PCR primers and index primers. The PCR products were purified with the AMpure XP kit and the quality of the libraries was evaluated on the Agilent Bioanalyzer 2100 (Agilent technologies, CA, USA). Clustering of the index-coded samples was done on a cBot Cluster Generation System using a TruSeq PECluster Kit 3-cBot-HS (Illumina Inc., CA, USA). Finally, the prepared samples were sequenced on Illumina HiSeq 4000 platform with 150 bp paired-end (PE) reads (see Additional file 3: Table S3 for an overview of number of raw reads per treatment).

### Transcriptome assembly

The sequencing reads were quality checked using FastQC v.0.10.1 [76]. Adapter sequences were removed and quality-filtered using Trimmomatic v.0.36 [77], with parameters set: ILLUMINACLIP: *file.fq*:2:30:10, LEADING: 5, TRAILING: 5, SLIDINGWINDOW:4:20, and removing all reads of less than 36 bp in length.

Before transcriptome assembly the raw reads were checked for potential contamination and remaining adapter sequences using local BLAST (NCBI-BLAST+ v.2.6.0 [37] was used for all local BLAST searches) against the UniVec database (ftp.ncbi.nlm.nih.gov/pub/UniVec/, accessed: 01 February 2018) using BLASTN with parameters: -reward 1 –penalty 5 –gapopen 3 –gapextend 3 –dust yes –soft_masking true –evalue 700 –searchsp 1,750,000,000,000. Raw reads were submitted to the NCBI SRA database under accession number PRJNA477295.Reads were assembled de novo using Trinity v.2.5.1 [32] using PE mode and without in silico read normalization.

The assembled transcripts were checked for contamination using DeconSeq v.0.4.3 [78] with a coverage of 90% and an alignment identity threshold of 98% against a set of bacterial, human, *B. oleracea*, *Z. mays,* and *N. tabacum* reference sequence sets. Sequence reference sets were obtained from various sources: the bacterial set is based on 1544 full representative ‘RefSeq’ bacterial genomes, from https://www.ncbi.nlm.nih.gov/assembly, accessed 24 May 2018; the Human Reference genome (GRCh38.p12) from ftp:// ftp.ncbi.nlm.nih.gov/genomes/Homo_sapiens/Assembled_chromosomes/seq/, accessed 24 May 2018; and the three host plant sets were all obtained from the NCBI genome database (*B. oleracea*, from ftp://ftp.ncbi.nih.gov/genomes/Brassica_oleracea/RNA/Gnomon_mRNA.fsa.gz; *N. tabacum*, from ftp://ftp.ncbi.nih.gov/genomes/Nicotiana_tabacum/RNA/Gnomon_mRNA.fsa.gz; and *Z. mays*, from ftp://ftp.ncbi.nih.gov/genomes/Zea_mays/RNA/Gnomon_mRNA.fsa.gz, all three accessed on 20 June 2018). Moreover, the assembly was cross-checked against various insect genomes: *Drosophila melanogaster* (ftp: //ftp.flybase.net/genomes/Drosophila_melanogaster/dmel_r6.21_FB2018_02/fasta/, accessed 05 June 2018), *B. mori* (ftp://ftp.ensemblgenomes.org/pub/metazoa/release-39/fasta/bombyx_mori/dna, accessed 05 June 2018) and *S. litura* (ASM27686v1; ftp://ftp.ncbi.nih.gov/genomes/Spodoptera_litura/Assembled_chromosomes/seq/, accessed 05 June 2018). Suspicious transcripts were subsequently removed. The final reference transcriptome was submitted to the NCBI TSA database and is available under accession GGRZ00000000, version GGRZ01000000 is used in this study.

Completeness of the final reference transcriptome assembly was accessed using BUSCO v.3.0.2 using the *Insecta_odb9* set for comparison [33].

### Transcript expression quantification

To estimate transcript expression, reads of all samples from each treatment were separately mapped to the reference transcriptome using Bowtie2 v.2.3.4 [79]. The isoform and gene abundance estimation was done using RSEM v.1.3.0 [80], resulting in a per-treatment count matrix which was fed into the Trinity package perl script ‘*abundance_estimates_to_matrix.pl’* to construct a raw (non-normalized) count matrix.

The raw count matrix was filtered by abundance based on count-per-million values (CPM; to account for library size differences between samples) using edgeR v.3.20.8 [81] in R v.3.4.3. Only genes with a minimum of 10 counts in at least two of the samples were considered expressed and retained in the dataset. Moreover, the filtered count matrix was cross-sample normalised using the ‘calcNormFactors’ function in edgeR using trimmed mean of M values (TMM) [82]. See Additional file 9: Table S9 for the CPM, TMM cross-sample normalised and filtered count matrix, and Additional file 10: Table S10 for the subset of DE genes. Venny v.2.1 [83] was used to evaluate gene similarity across the diet treatments using the filtered and normalised gene matrix (Venn diagram is provided in Additional file 11: Figure S1, results are presented in Additional file 12: Data S1). To estimate sample and biological replicate relationships, the filtered and normalised count matrix was analysed using cluster bootstrapping with 10,000 replications with the R package PVClust v.2.0 [34].

The differential expression analysis was performed using DESeq2 v.1.18.1 [35] as implemented in the Trinity package under default settings; min_rowSum_counts = 2 and using the median of ratios method for normalization, taking into account sequencing depth and RNA composition. Transcripts were considered significantly differentially expressed with a minimal fold-change of four between any of the treatments and a false discovery rate (FDR) of *p*-value ≤1e-3. The CPM and TMM normalised expression values of all differentially expressed transcripts were hierarchically clustered and cut at 50% using the Trinity provided script ‘*define_clusters_by_cutting_tree.pl*’. This resulted in six clusters of differentially expressed transcripts with similar expression patterns that were used in the cluster-specific GO analysis.

### Transcript annotation

The sequences, on isoform level, were annotated using the Trinotate pipeline v.3.0 [36]. TransDecoder v.5.0.2 from the Trinity package was used to identify candidate coding regions within the assembled transcripts [36], identifying open reading frames (ORFs) with a minimal length of 100 amino acids by default. The TransDecoder-predicted protein sequences were used as a query for a BLASTP search using the manually annotated and non-redundant Swiss-Prot database (ftp://ftp.uniprot.org/pub/databases/uniprot/current_release/knowledgebase/complete/uniprot_sprot.dat.gz; release 2018_06, accessed June 2018). The transcripts from the assembly were used as a query for a BLASTX search using the same protein database. Moreover, a protein domain search was performed using ‘*hmmscan*’ from HMMER v.3.1b2 against the Pfam-A database v.31. Signal peptides were predicted using SignalP 4.1 server [41] and TMHMM v.2.0 was used for transmembrane region prediction [42]. See Additional file 4: Table S4 for the Trinotate-pipeline derived annotations. Additionally, a local BLASTX search (settings: max_hsps of 1, best_hit_overhang set on 0.1, E-value cutoff ≤1e-3) was conducted using the assembled transcripts as a query against a database consisting of all local Arthropoda protein sequences downloaded from the NCBI protein database (accessed, February 2018).

Gene ontology (GO) analysis was performed using the GO annotations from the Trinotate pipeline. GO terms, including all ancestral terms were extracted from the annotation report. The R package GOseq v.1.30.0 was used for the analysis, which takes gene length bias into account [43]. Only statistically overrepresented GO terms were taken into account that had an adjusted *p*-value < 0.05, according to Benjamini-Hochberg (BH) correction for multiple hypothesis testing [44]. For each of the six identified DE clusters the statistically overrepresented GO terms (BH corrected p-value < 0.05) were calculated relative to the reference of all other GO annotated transcripts of the reference assembly. The retrieved significant GO terms were compiled into broader terms, generic GOslim categories (subset used: goslim_generic.obo, the Gene Ontology Consortium; accessed August 2018), using the R package GOstats v.2.44.0 [84].

The reference transcriptome was screened for genes of five major gene families known to be involved in toxin detoxification: P450, CCE, GST, UGT and ABC transporters. Annotated genes from five detoxification gene families from Lepidoptera members of the Noctuidae, Plutellidae, Sphingidae and Bombycidae were combined in a local protein BLAST database. The database consisted of 2369 identified protein sequences derived from the following eight species: the fall armyworm (*S. frugiperda*) (both corn and rice populations, OGS2.2 [14];), tobacco cutworm (*S. litura*) (protein set 002706865.1 genome ASM270686v1, from NCBI Genome database [10];), the beet armyworm (*S. exigua*) (all P450 and UGT genes [18];), the silkworm (*Bombyx mori*) (protein set 000151625.1 genome ASM15162v1, from NCBI Genome database; International Silkworm Genome [85]), the diamondback moth (*Plutella xylostella*) (OGSv2 [86];), the tobacco hornworm (*Manduca sexta*) (OGS2 [23];), and both the cotton bollworm (*Helicoverpa armigera*) and corn earworm (*Helicoverpa zea*) [8]. Transcripts from the reference assembly were run as query in a BLASTX run against the created detoxification protein database. For each group of isoforms the highest expressed transcript was selected as gene representative for the detoxification-gene annotation. Only BLAST hits with an E-value >1e^− 5^ were selected as putative detoxification genes.

## Supplementary information

**Additional file 1: Table S1.** Overview of the results of the feeding assays.

**Additional file 2: Table S2.** Overview feeding assay statistics.

**Additional file 3: Table S3.** Overview of the number of raw reads per library and the final number of reads after trimming, cleaning and contamination checks.

**Additional file 4: Table S4.** Trinotate annotation report.

**Additional file 5: Table S5.** BLASTX annotated DE genes using local Arthropoda database.

**Additional file 6: Table S6.** overview of all GO slims per cluster.

**Additional file 7: Table S7.** BLASTX annotated DE genes using the local detoxification gene database.

**Additional file 8: Table S8.** RNA concentrations for all samples.

**Additional file 9: Table S9.** CPM, TMM cross-sample normalised and filtered count matrix.

**Additional file 10: Table S10.** CPM, TMM cross-sample normalised and filtered count matrix only including DE genes.

**Additional file 11: Figure S1.** Venn diagram indicating the number of expressed transcripts shared or unique in *Spodoptera exigua* larvae feeding on different diet treatments based on the filtered and normalized count matrix. Larvae developed on *Zea mays, Brassica oleracea*, *Nicotiana tabacum* or artificial diet until reaching the third larval stage. The total number of transcripts included was 58,749.

**Additional file 12: Data S1.** Additional results of transcriptome assembly statistics and annotation.

## Data Availability

All material is submitted to the appropriate NCBI databases (see text). Available under BioProject accession number PRJNA477295 (https://www.ncbi.nlm.nih.gov/bioproject/). Genetic voucher material is stored in − 80 °C storage facility of the Biosystematics Group, Wageningen University & Research.

## Abbreviations

ABC: ATP-binding cassette transporters
BP: GO category - biological processes
CCE: Carboxyl/Choline esterase
CPM: Count per million
DE: Differentially expressed
GST: Glutathione-S-transferase
MF: GO category - molecular functions
P450: Cytochrome P450 monooxygenase
TMM: Trimmed mean of M values
UGT: UDP-glycosyltransferase
