# Supplementary figures and images for: An influential meal: host plant dependent transcriptional variation in the beet armyworm, Spodoptera exigua (Lepidoptera: Noctuidae)
Source: BMC Genomics. 2019 Nov 13;20:845. doi: 10.1186/s12864-019-6081-7 (PMC6854893; doi:10.1186/s12864-019-6081-7)

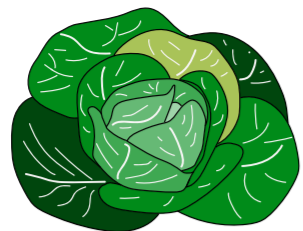

*B. oleracea*

*Z. mays*

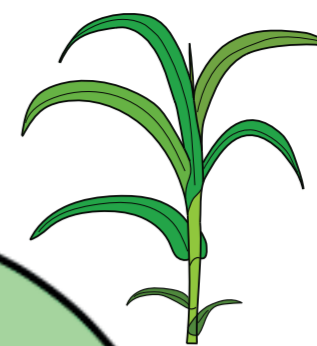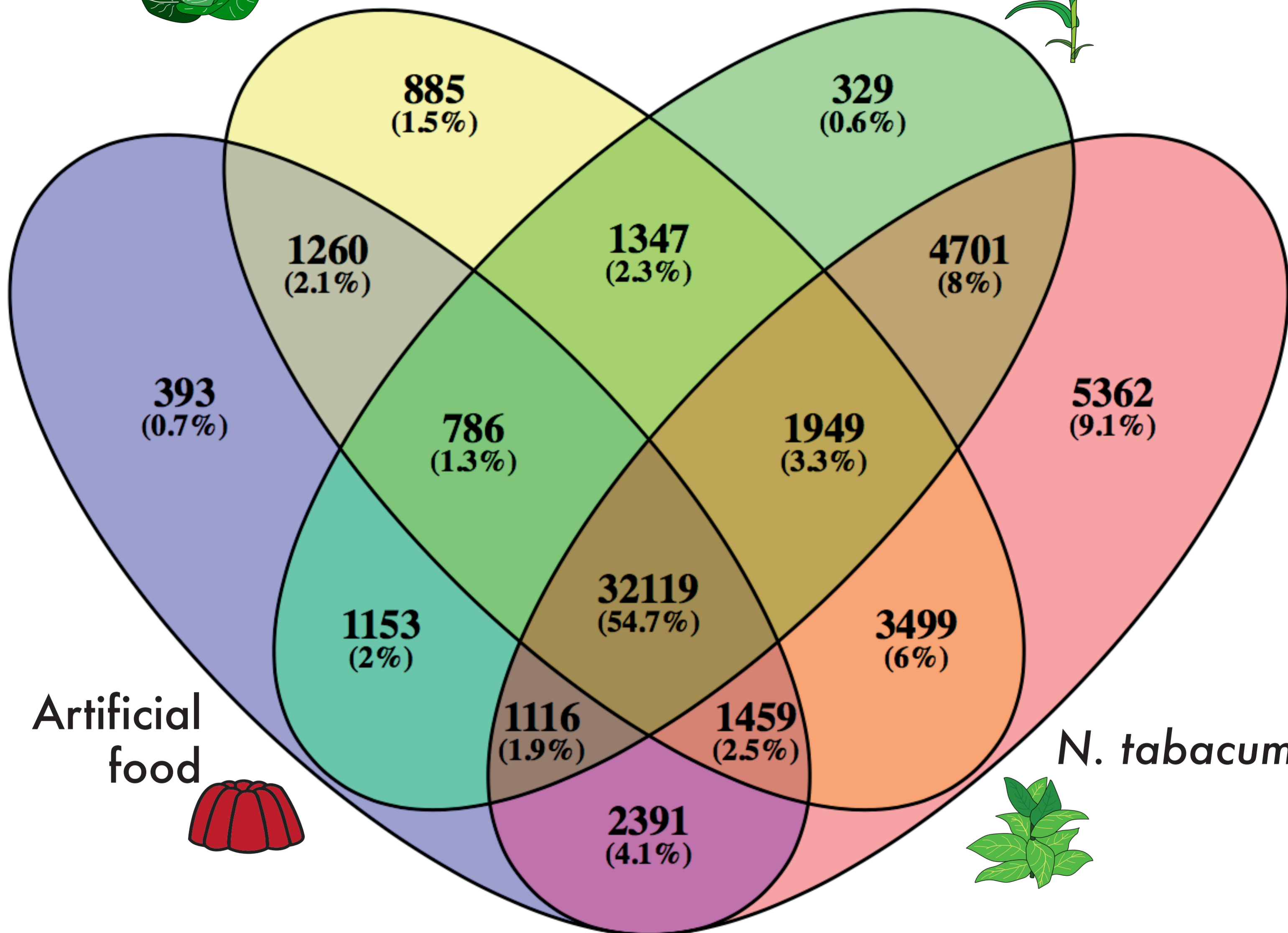

Artificial  
food

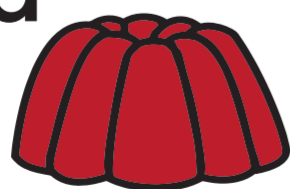

*N. tabacum*

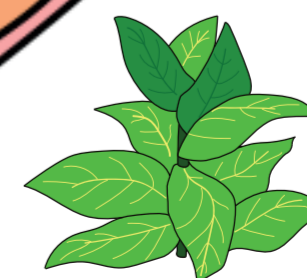

Supplement: Supplementary file 11 — Additional file 11: Figure S1. Venn diagram indicating the number of expressed transcripts shared or unique in Spodoptera exigua larvae feeding on different diet treatments based on the filtered and normalized count matrix. Larvae developed on Zea mays, Brassica oleracea, Nicotiana tabacum or artificial diet until reaching the third larval stage. The total number of transcripts included was 58,749. [file 12864_2019_6081_MOESM11_ESM.pdf]
