# Supplementary material for: An influential meal: host plant dependent transcriptional variation in the beet armyworm, Spodoptera exigua (Lepidoptera: Noctuidae)
Source: BMC Genomics. 2019 Nov 13;20:845. doi: 10.1186/s12864-019-6081-7 (PMC6854893; doi:10.1186/s12864-019-6081-7)
Supplement: Supplementary file 12 — Additional file 12: Data S1. Additional results of transcriptome assembly statistics and annotation. [file 12864_2019_6081_MOESM12_ESM.docx]

Supplementary data file 1 – Results

**Results**

**Transcriptome assembly statistics**

Of the total 1,070,425,478 raw Illumina reads from 12 samples, 982,100,776 Illumina reads were used for the assembly after an initial filtering and vector contamination check. The final cleaned de novo reference assembly contained 251,722 isoforms and 210,101 transcripts on gene level.

The final assembly contained a total number of 195,502,771 bases and an average length of 776.66 bases per transcript.

**First evaluation of gene similarity between treatments**

Venny v.2.1 [1] was used to evaluate gene similarity across the diet treatments using the filtered and normalised gene matrix (Venn diagram is provided in Supplementary Figure 1).

Based on the filtered and normalised count matrix, comprising 58,749 transcripts, 32,119 were transcriptionally active in all four different diet treatments. All diet treatments showed unique transcripts. For larvae feeding on Z. mays, 329 transcripts were unique, the least of all treatments. In total 393 transcripts were unique for larvae feeding on artificial diet, 885 for B. oleracea and 5,362 transcripts were unique for N. tabacum fed larvae.

**Transcript annotation**

The reference transcriptome assembly was annotated using the Trinotate pipeline v.3.0 [2]. The pipeline consisted of both a BLASTP and BLASTX search, using local BLAST (NCBI-BLAST+ v.2.6.0 [3]) against the manually annotated and non-redundant Swiss-Prot database [4], including GO, KEGG and EggNOG annotations. Further, protein domain searches were performed using HMMER v.3.1b2 [5] against the Pfam-A database [6], signal peptide detection using the SignalP 4.1 server [7] and a transmembrane region prediction using TMHMM server v.2.0 [8].

A total of 45,435 isoforms were annotated using BLASTX against the Swiss-Prot database. The BLASTP search of the translated protein sequences against the same database resulted in 27,751 annotations. Furthermore, the Trinotate pipeline annotated signal peptides in 3,648 transcripts, 6,447 transcripts with transmembrane regions and protein domains were identified in 26,142 transcripts. Finally, for 44,216 isoforms at least one GO-term was identified while this was 36,469 for KEGG- and 34,384 for COG-terms.

In addition to the Trinotate annotation a local BLASTX search was conducted using all DE transcripts as a query against a local database containing all Arthropoda protein sequences as retrieved from the NCBI protein database. In total 1,484 DE transcripts were annotated.

**References**

1. Oliveros JC. **VENNY. An interactive tool for comparing lists with Venn Diagrams**. 2007.

2. Haas BJ, Papanicolaou A, Yassour M, Grabherr M, Blood PD, Bowden J, Couger MB, Eccles D, Li B, Lieber M. **De novo transcript sequence reconstruction from RNA-seq using the Trinity platform for reference generation and analysis**. *Nature protocols* 2013, **8**(8):1494-1512.

3. Camacho C, Coulouris G, Avagyan V, Ma N, Papadopoulos J, Bealer K, Madden TL. **BLAST+: architecture and applications**. *BMC bioinformatics* 2009, **10**(1):421.

4. Apweiler R, Bairoch A, Wu CH, Barker WC, Boeckmann B, Ferro S, Gasteiger E, Huang H, Lopez R, Magrane M. **UniProt: the universal protein knowledgebase**. *Nucleic acids research* 2004, **32**:115-119.

5. Team HD. **Search sequence(s) against a profile database**. 2013.

6. Finn RD, Coggill P, Eberhardt RY, Eddy SR, Mistry J, Mitchell AL, Potter SC, Punta M, Qureshi M, Sangrador-Vegas A. **The Pfam protein families database: towards a more sustainable future**. *Nucleic acids research* 2015, **44**(D1):D279-D285.

7. Petersen TN, Brunak S, von Heijne G, Nielsen H. **SignalP 4.0: discriminating signal peptides from transmembrane regions**. *Nature methods* 2011, **8**(10):785.

8. Krogh A, Larsson B, Von Heijne G, Sonnhammer EL. **Predicting transmembrane protein topology with a hidden markov model: application to complete genomes1**. *Journal of molecular biology* 2001, **305**(3):567-580.
